# Supplementary material for: Clinical Characterization and Founder Effect Analysis in Chinese Patients with Phospholipase A2-Associated Neurodegeneration
Source: Brain Sci. 2022 Apr 19;12(5):517. doi: 10.3390/brainsci12050517 (PMC9138368; doi:10.3390/brainsci12050517)
Supplement: Supplementary file 1 [file brainsci-12-00517-s001.zip › brainsci-1650042-supplementary/Supplementary files (revised 2)/Figure S1.pdf]

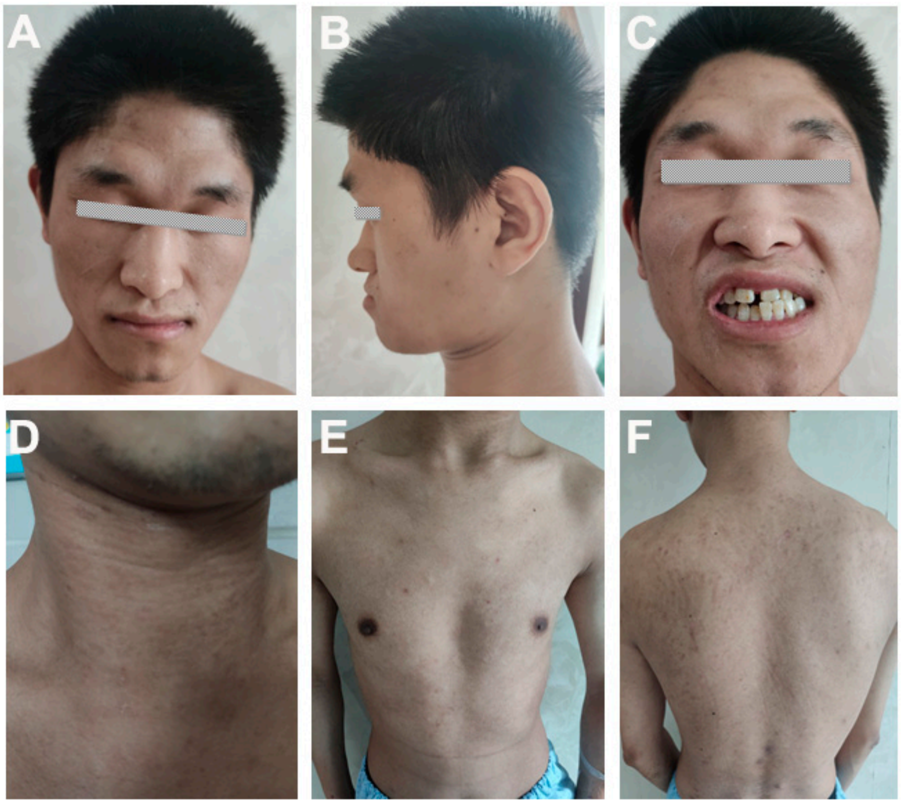

Figure S1. Special clinical features of the Case 2. (A-C) Special face: high eyebrow arches, deep eye sockets, abnormal tooth arrangement, and protruding jaw; (D-F) skin hyperpigmentation and rash over whole body, scoliosis.
